# Index Der Deutschen Zahnärztlichen Literatur Und Zahnärztliche Bibliographie

**Published:** 1904-07

**Authors:** 


### Bibliography.

INDEX DER DEUTSCHEN ZAHNÄRZTLICHEN LITERATUR UND ZAHNÄRZTLICHE BIBLIOGRAPHIE. Bearbeitet von Prof. Dr. Port in Heidelberg. Hörning und Berkenbusch, Heidelberg, 1904.

This very important work which, as the title implies, covers the German dental literature and the bibliography of dentistry, the latter in part only, as the author has confined the notices of the work of foreign writers to the brief abstract translations into German found in the periodical literature of those speaking and writing that language. This makes the book imperfect so far as the dental literature of the world is concerned.

This "Index" had its origin at the annual meeting of the Central Association of German Dentists, held at Munich, 1902, at which a commission was appointed to prepare an index of dental literature. The following were named to serve in that capacity: Julius Parreidt, Leipzig; Schäffer-Stuckert, Frankfort a/M.; Sternfeld, Munich; Professor Witzel, Essen; and the author, Professor Port.

The Index covers one hundred and eighty pages, the subject-matter being divided into the various branches practised in dentistry. This includes not only elaborate works, but papers as they have appeared in various periodicals. This has involved an enormous amount of labor upon the part of the author. The patient research made necessary to perfect this deserves the special thanks of the author's co-workers among German-speaking peoples.

The book has, of course, but a limited value for those who confine their reading to English. The intention of the author not to enter thoroughly into foreign work has necessarily reduced his notices of English and American writings. This seems unfortunate, as the author's ability and industry applied to all countries would have made this the most valuable book of reference for the literature of dentistry ever published. As it is, the English reader must wait, it is feared, a long period for some industrious compiler to prepare this much-needed work.

On carefully scanning the pages of this book one is struck with the fact that much of minor importance finds a place there, and much of real value, in the world's work, is omitted. It is rather surprising that Black's labors have not found any notice in German periodical literature; at least, our author makes no mention of his name. Nothing has been said of Farrar, Angle, and Talbot in Orthodontia, or of the latter in his elaborate work on "Interstitial Gingivitis." This means that not only these books, but many others of equal value, are unknown to German readers. Upon the other hand, the long list of valuable works printed in German are absolutely unknown to American readers, beyond, in some instances, a mere abstract of contents. Until all readers become linguists, or the world has a universal language for scientific publications, this ignorance must continue to produce a narrow conception of the work of each branch of science in foreign tongues. Many books have been published in German in recent years, of very great value, but so far these have not found translators, consequently those who might materially benefit by the reading are shut out entirely from large stores of knowledge. All this may be changed in the not far distant future, through greater attention paid in the schools to the study of modern languages. This has become imperative through the constant intermingling of nationalities. In the mean time the writer would express the hope that in the near future some American would follow the good example of Professor Port and prepare an index of American writings and American authors, such as we find in this volume devoted to German dentistry.

To those who can read German this Index will prove of great value as a work of reference, and it can be recommended to fill worthily a place in all libraries.

